# Hyperthyroidism Is Genetically Associated With Reduced Risk of Parkinson's Disease: A Mendelian Randomization Analysis

**DOI:** 10.1111/ejn.70616

**Published:** 2026-07-08

**Authors:** Meng Luo, Shiren Huang, Wei Wang

**Affiliations:** ^1^ Department of Neurology Bazhong Traditional Chinese Medicine Hospital (Bazhou District People's Hospital of Bazhong City) Bazhong Sichuan Province China; ^2^ Jiading Branch of Shanghai General Hospital Shanghai Jiao Tong University School of Medicine Shanghai China; ^3^ Department of Neurology Chongqing Sanbo Jiangling Hospital Chongqing China

**Keywords:** Graves' disease, Mendelian randomization, Parkinsons' disease, thyroid dysfunction, thyrotoxicosis

## Abstract

Parkinson's disease (PD) is a progressive neurodegenerative disorder whose aetiology involves an intricate interplay of genetic, immune, metabolic and environmental factors. Endocrine dysfunction—particularly disturbances of thyroid hormone signalling—has been proposed as a contributor to neurodegeneration, but conventional observational studies have produced inconsistent results, and prior Mendelian randomization (MR) work has largely focused on continuous thyroid biomarkers rather than clinically defined hyperthyroid disease states. To clarify this relationship, we performed a two‐sample bidirectional and multivariable MR (MVMR) analysis using large‐scale genome‐wide association study (GWAS) summary statistics from the FinnGen and IEU Open GWAS databases (European ancestry). Single‐nucleotide polymorphisms (SNPs) reaching genome‐wide significance (*p* < 5 × 10^−8^) for Graves' disease and thyrotoxicosis with diffuse goitre served as instrumental variables. The inverse‐variance weighted (IVW) method was the primary analysis, complemented by MR–Egger, weighted median, weighted mode and simple mode estimators, and MVMR adjusted for smoking, alcohol consumption, and body mass index (BMI). In forward analyses, genetically proxied Graves' disease (OR = 0.942, 95% CI 0.901–0.985, *p* = 0.008) and thyrotoxicosis with diffuse goitre (OR = 0.929, 95% CI 0.879–0.982, *p* = 0.009) were associated with a lower risk of PD, whereas reverse analyses showed no significant effect of genetic liability to PD on either thyroid trait. The inverse associations remained stable across MVMR models, and sensitivity analyses (Cochran's *Q*, MR–Egger intercept, MR‐PRESSO, leave‐one‐out) showed no evidence of heterogeneity or horizontal pleiotropy. Collectively, these findings provide genetic evidence consistent with a protective relationship between hyperthyroid disease states and PD, independent of major lifestyle confounders. By focusing on clinically defined hyperthyroid entities rather than continuous thyroid indices, our study complements prior MR work and highlights the thyroid–brain axis—encompassing thyroid hormone signalling and autoimmune‐mediated immune modulation—as a biologically plausible and potentially modifiable contributor to PD risk that warrants further mechanistic and translational investigation.

## Graphical abstract

This bidirectional and multivariable Mendelian randomization study found that genetically predicted Graves' disease and thyrotoxicosis with diffuse goitre were associated with a reduced risk of Parkinson's disease. These inverse associations remained stable after adjustment for smoking, alcohol consumption and BMI, with no evidence of reverse causality or horizontal pleiotropy. The findings highlight the thyroid–brain axis as a potential contributor to Parkinson's disease susceptibility.

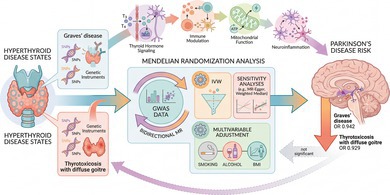

**Abbreviations**
BMI: body mass index
CI: confidence interval
FT4: free thyroxine
GWAS: genome‐wide association study
ICD: International Classification of Diseases
IV: instrumental variable
IVW: inverse‐variance weighted
LD: linkage disequilibrium
MR: Mendelian randomization
MR‐PRESSO: Mendelian Randomization Pleiotropy RESidual Sum and Outlier
MVMR: multivariable Mendelian randomization
OR: odds ratio
PD: Parkinson's disease
SM: simple mode
SNP: single‐nucleotide polymorphism
T₃: triiodothyronine
T₄: thyroxine
TSH: thyroid‐stimulating hormone
WM: weighted mode
WME: weighted median estimator

## 1 Introduction

Parkinson's disease (PD) is a progressive neurodegenerative disorder characterized by motor symptoms such as tremor, rigidity and bradykinesia, alongside a wide range of nonmotor manifestations (Bloem et al. 2021). It is one of the fastest‐growing neurological conditions globally and imposes substantial medical and socioeconomic burdens (Dorsey and Bloem 2018; GBD 2016 Parkinson's Disease Collaborators. 2018). Despite extensive research, the aetiology of PD remains incompletely understood and involves a complex interplay of genetic, environmental and metabolic factors (Kalia and Lang 2015). Increasing evidence suggests that endocrine dysfunction, particularly that involving thyroid hormones, may contribute to PD pathogenesis through shared molecular and inflammatory pathways. Hyperthyroid disorders—including Graves' disease and thyrotoxicosis with diffuse goitre (Chaker et al. 2017)—are among the most prevalent endocrine diseases worldwide. Thyroid hormones play crucial roles in neuronal differentiation, synaptic plasticity and mitochondrial metabolism (Morte and Bernal 2014), and dysregulated thyroid function has been implicated in oxidative stress, neuroinflammation and dopaminergic vulnerability—mechanisms also central to PD pathology (Cano‐Europa et al. 2008; Rahaman et al. 2001). Observational studies have, however, reported inconsistent associations between thyroid dysfunction and PD risk (Chen et al. 2020), with some suggesting an increased risk of PD in patients with hypothyroidism and others finding no significant relationship (Berger and Kelley 1981). Such discrepancies likely reflect residual confounding, reverse causation and methodological heterogeneity inherent to traditional epidemiological designs.

Mendelian randomization (MR) offers a robust framework for inferring causality by using genetic variants as instrumental variables (IVs) for modifiable exposures. By exploiting the random allocation of alleles at conception, MR minimizes confounding and reverse causation and provides a more reliable estimate of exposure–outcome relationships (Davies et al. 2018). Recent advances in large‐scale genome‐wide association study (GWAS) have enabled MR analyses of thyroid–brain interactions (Burgess et al. 2020; Lovegrove et al. 2024). Several recent MR studies have already examined continuous thyroid biomarkers in relation to PD and other neurological phenotypes: Zeng and colleagues reported no causal link between thyroid‐stimulating hormone (TSH) or free thyroxine and PD in a bidirectional MR framework (Zeng et al. 2024); Wang and colleagues observed associations of thyroid function indices with prospective memory and dementia, but only limited signals with PD (Wang et al. 2025); and Lei and colleagues, using bidirectional MR with co‐localization, suggested a protective effect of PD liability on hypothyroidism rather than the reverse direction (Lei et al. 2024). A systematic review of the broader literature further highlighted heterogeneous links between thyroid dysfunction and a spectrum of movement disorders, with relatively sparse high‐quality data (Schneider et al. 2023). Notably, these prior MR efforts have largely focused on continuous thyroid indices rather than clinically defined hyperthyroid disease states, in which excess thyroid hormone signalling coexists with autoimmune or structural thyroid abnormalities; this disease‐level exposure has not been systematically interrogated with respect to PD.

In the present study, we conducted a bidirectional and multivariable Mendelian randomization (MVMR) analysis to investigate the causal associations between two clinically defined hyperthyroid disease entities—Graves' disease and thyrotoxicosis with diffuse goitre—and the risk of PD. Summary‐level GWAS data were obtained from the FinnGen and IEU Open GWAS databases, restricted to European‐ancestry populations to minimize population stratification bias (Bycroft et al. 2018). We further adjusted for potential lifestyle confounders, including smoking, alcohol consumption and body mass index (BMI), within MVMR models (Davies et al. 2018). By combining rigorous sensitivity analyses with complementary MR estimators, our study aims to provide a disease‐centred genetic perspective on the thyroid–brain axis in PD and to inform future mechanistic and preventive research.

## 2 Materials and Methods

### 2.1 Data Sources

Summary‐level GWAS data for hyperthyroid disorders—comprising Graves' disease and thyrotoxicosis with diffuse goitre—were obtained from the FinnGen database (https://www.finngen.fi/en) and the IEU Open GWAS database (https://gwas.mrcieu.ac.uk/datasets/). GWAS summary statistics for PD comprised 13,358 cases and 43,071 controls, with a total of 17,891,936 single‐nucleotide polymorphisms (SNPs). All participants in the included GWAS datasets were of European ancestry, in order to minimize bias from population stratification, ethnic heterogeneity and geographical confounding. Details of the included datasets are summarized in Table S1.

FinnGen and the IEU Open GWAS database were selected for several methodological reasons. Both consortia provide large, harmonized and quality‐controlled summary statistics derived from population‐based cohorts with deep clinical phenotyping. FinnGen offers high‐resolution ICD‐based endpoint definitions for endocrine disorders, including separate curated phenotypes for Graves' disease and thyrotoxicosis with diffuse goitre, which are essential for disease‐level rather than biomarker‐level MR. The IEU Open GWAS platform aggregates the most widely used PD summary statistics in standardized formats with consistent metadata, facilitating reproducible bidirectional and multivariable analyses. Restricting both exposure and outcome data to European‐ancestry datasets from these complementary repositories reduces ancestry‐related artefacts and improves comparability with previous MR studies of the thyroid–PD relationship (Wang et al. 2025; Zeng et al. 2024; Lei et al. 2024). We separately searched East Asian and trans‐ancestry repositories (BBJ, GBMI) for Graves' disease and thyrotoxicosis but did not identify summary statistics that met the methodological prerequisites for valid MR (sufficient genome‐wide significant instruments with publicly available effect sizes and quality‐controlled standard errors); the implications of this restriction are discussed below.

### 2.2 Selection of IVs

To identify valid IVs, all SNPs associated with the exposures were selected at a genome‐wide significance threshold of *p* < 5 × 10^−8^. To avoid bias arising from linkage disequilibrium (LD) between SNPs, we applied clumping parameters of *r* ^2^ < 0.001 and a genetic distance of 10,000 kb, ensuring genetic independence among IVs. The strength of each IV was assessed using the *F*‐statistic, calculated as *F* = [(*N* − *K* − 1)/*K*] × [*R* ^2^/(1 − *R* ^2^)], where *N* represents the sample size, *K* the number of IVs and *R* ^2^ the proportion of variance in the exposure explained by the IVs. All selected SNPs had *F* values greater than 10, indicating that weak‐instrument bias was unlikely.

### 2.3 MR Analyses

Bidirectional MR analyses were conducted to evaluate the causal relationship between thyroid diseases and PD. The inverse‐variance weighted (IVW) method was used as the primary analytical approach to estimate causal effects. Additional MR methods—including the weighted median estimator (WME), weighted mode (WM), simple mode (SM) and MR–Egger regression—were applied as complementary sensitivity analyses.

### 2.4 MVMR

To account for potential confounding factors, we performed MVMR analyses using the MVMR‐IVW method. The MVMR–Egger intercept and corresponding *p*‐value were calculated to assess horizontal pleiotropy. In these models, smoking, alcohol consumption and BMI were included as covariates to adjust for their potential confounding effects. The aim was to estimate the direct causal effect of thyroid diseases on PD risk, independent of these lifestyle and metabolic factors.

### 2.5 Sensitivity Analyses

Sensitivity analyses were performed to evaluate the reliability and robustness of MR results. Heterogeneity was assessed using Cochran's *Q* statistic, whereas horizontal pleiotropy was examined through the MR–Egger intercept and MR‐PRESSO tests. Visual inspection of the MR results was conducted using scatter plots, forest plots and funnel plots to assess the consistency and symmetry of causal estimates. Leave‐one‐out analyses were further conducted to confirm that no single SNP had a dominant effect on the overall causal estimates. For MVMR models, similar sensitivity analyses were carried out to evaluate heterogeneity and pleiotropy across all adjusted models.

## 3 Results

### 3.1 Selection and Validation of IVs

All IVs selected for the MR analyses reached genome‐wide significance (*p* < 5 × 10^−8^) and exhibited sufficient strength, with *F*‐statistics exceeding 10, indicating the absence of weak‐instrument bias. The detailed characteristics of the SNPs used as IVs are presented in Table S2, confirming their robustness and validity for subsequent causal inference analyses.

### 3.2 Causal Association Between Hyperthyroid Disorders and PD

**FIGURE 1:**
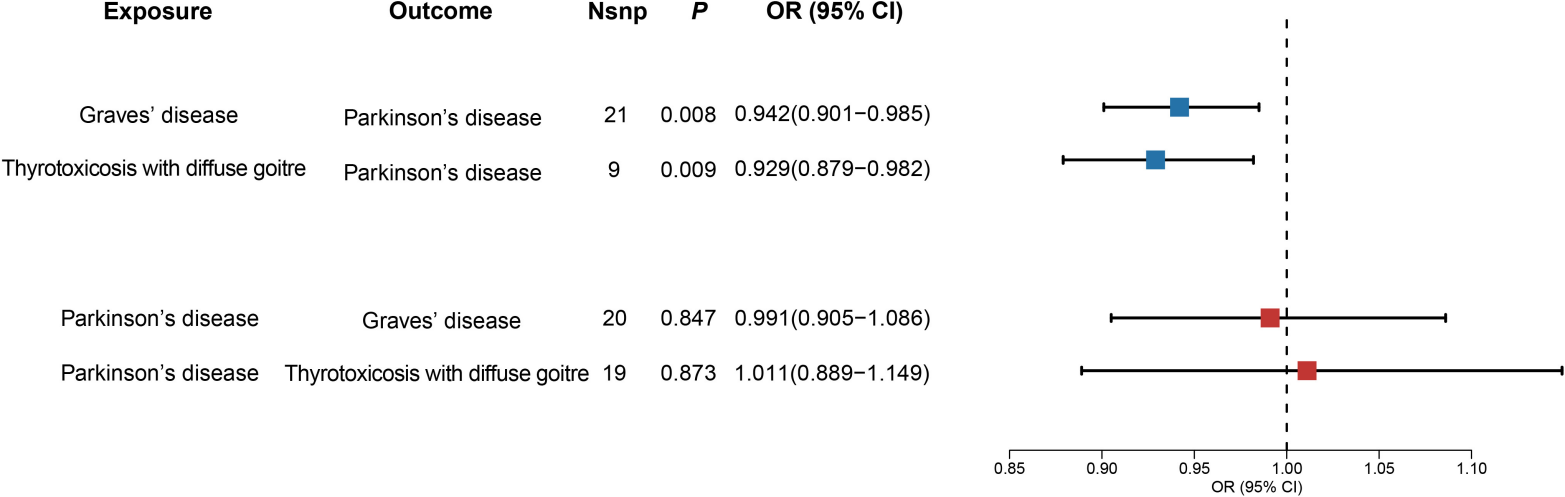
Bidirectional Mendelian randomization analysis between hyperthyroidism diseases and Parkinson's disease.

Bidirectional MR analyses were performed to explore the potential causal relationships between thyroid diseases and PD. In the forward MR analysis, genetically predicted thyroid disorders were significantly associated with a lower risk of PD. Specifically, IVW estimates showed that Graves' disease (OR = 0.942, 95% CI = 0.901–0.985, *p* = 0.008) and thyrotoxicosis with diffuse goitre (OR = 0.929, 95% CI = 0.879–0.982, *p* = 0.009) were each significantly associated with a reduced risk of developing PD. These consistent inverse associations are consistent with a protective effect of hyperthyroid conditions on PD risk (Figure 1).

In the reverse MR analyses, where PD was treated as the exposure and thyroid disorders as outcomes, no significant causal effects were observed for any of the thyroid diseases. The IVW estimates indicated that genetically predicted PD did not significantly influence the risk of Graves' disease or thyrotoxicosis with diffuse goitre (Figure 1). Together, these findings provide evidence consistent with a unidirectional inverse relationship in which genetic liability to hyperthyroid disease is associated with lower PD risk, but not vice versa.

### 3.3 MVMR Adjusted for Confounding Factors

**FIGURE 2:**
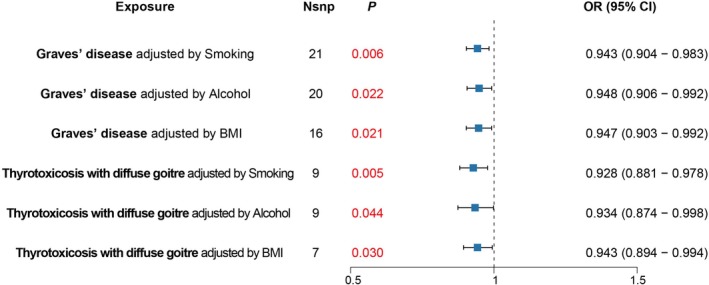
Multivariable Mendelian randomization analysis of hyperthyroidism diseases and Parkinson's disease adjusted for potential confounders (smoking, alcohol consumption and BMI).

To evaluate the stability of the causal associations after accounting for potential confounders, MVMR analyses were performed by including smoking, alcohol consumption and BMI as additional covariates (Figure 2). Across all MVMR models, the direction and magnitude of the causal estimates remained consistent with the primary univariable MR results, indicating that the protective association between genetically predicted hyperthyroidism and PD was robust and largely independent of lifestyle or metabolic factors.

After adjustment for smoking, Graves' disease was significantly associated with a reduced risk of PD (OR = 0.943, 95% CI = 0.904–0.983, *p* = 0.006), and thyrotoxicosis with diffuse goitre showed a similar protective effect (OR = 0.928, 95% CI = 0.881–0.978, *p* = 0.005).

When alcohol consumption was included as a covariate, both associations remained significant (Graves' disease: OR = 0.948, 95% CI = 0.906–0.992, *p* = 0.022; thyrotoxicosis with diffuse goitre: OR = 0.934, 95% CI = 0.874–0.998, *p* = 0.044).

Similarly, adjustment for BMI produced consistent results (Graves' disease: OR = 0.947, 95% CI = 0.903–0.992, *p* = 0.021; thyrotoxicosis with diffuse goitre: OR = 0.943, 95% CI = 0.894–0.994, *p* = 0.030).

Across all three MVMR models, the 95% confidence intervals did not cross the null, and the effect sizes remained narrowly distributed (OR ≈ 0.93–0.95), confirming a stable and statistically significant protective association between genetically predicted hyperthyroidism and PD risk.

### 3.4 Sensitivity and Robustness Analyses

**FIGURE 3:**
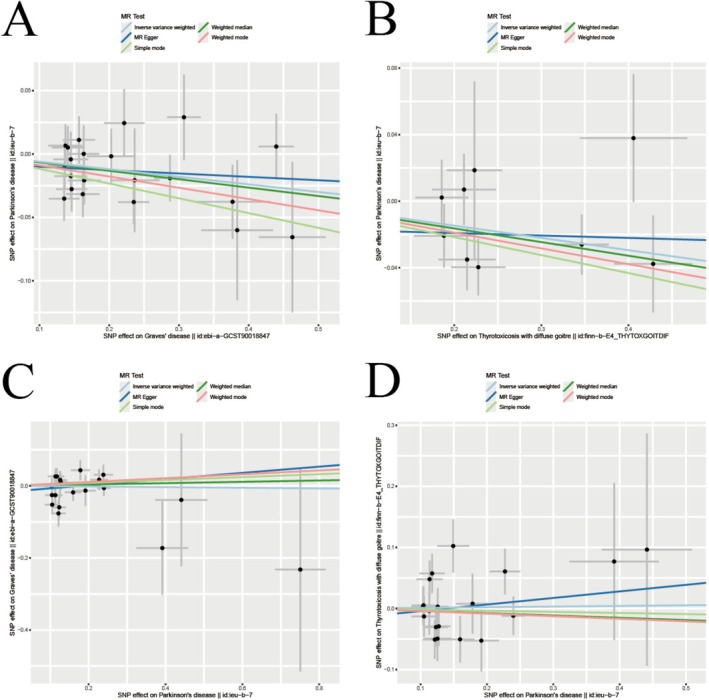
Scatter plots of bidirectional Mendelian randomization analyses. (A–B) represent forward MR analyses with exposures of Graves' disease and thyrotoxicosis with diffuse goitre, respectively; (C–D) represent reverse MR analyses with the corresponding outcomes.

**FIGURE 4:**
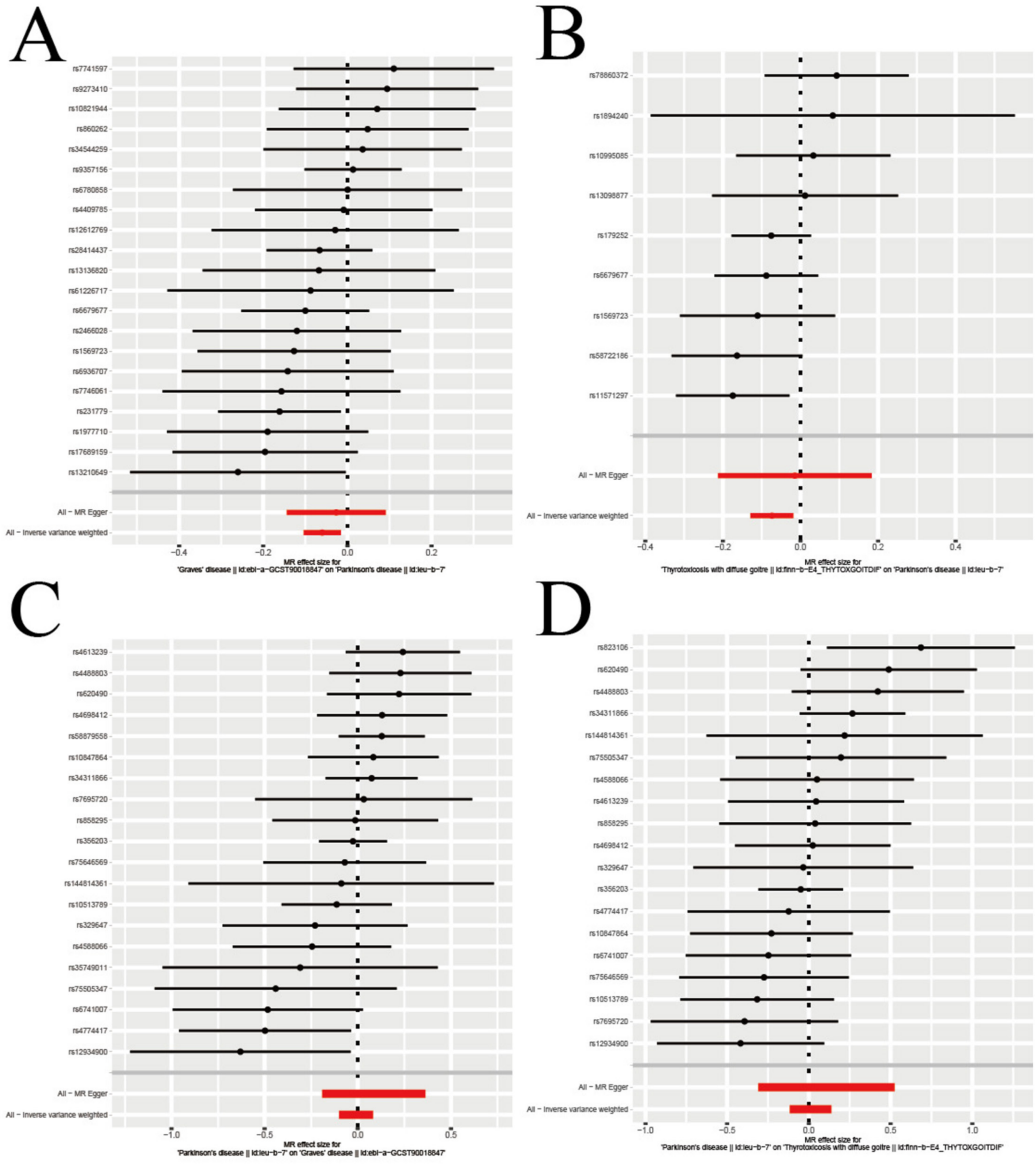
Forest plots of bidirectional Mendelian randomization analyses. (A–B) depict forward MR analyses for Graves' disease and thyrotoxicosis with diffuse goitre, respectively; (C–D) depict reverse MR analyses for the same outcomes.

**FIGURE 5:**
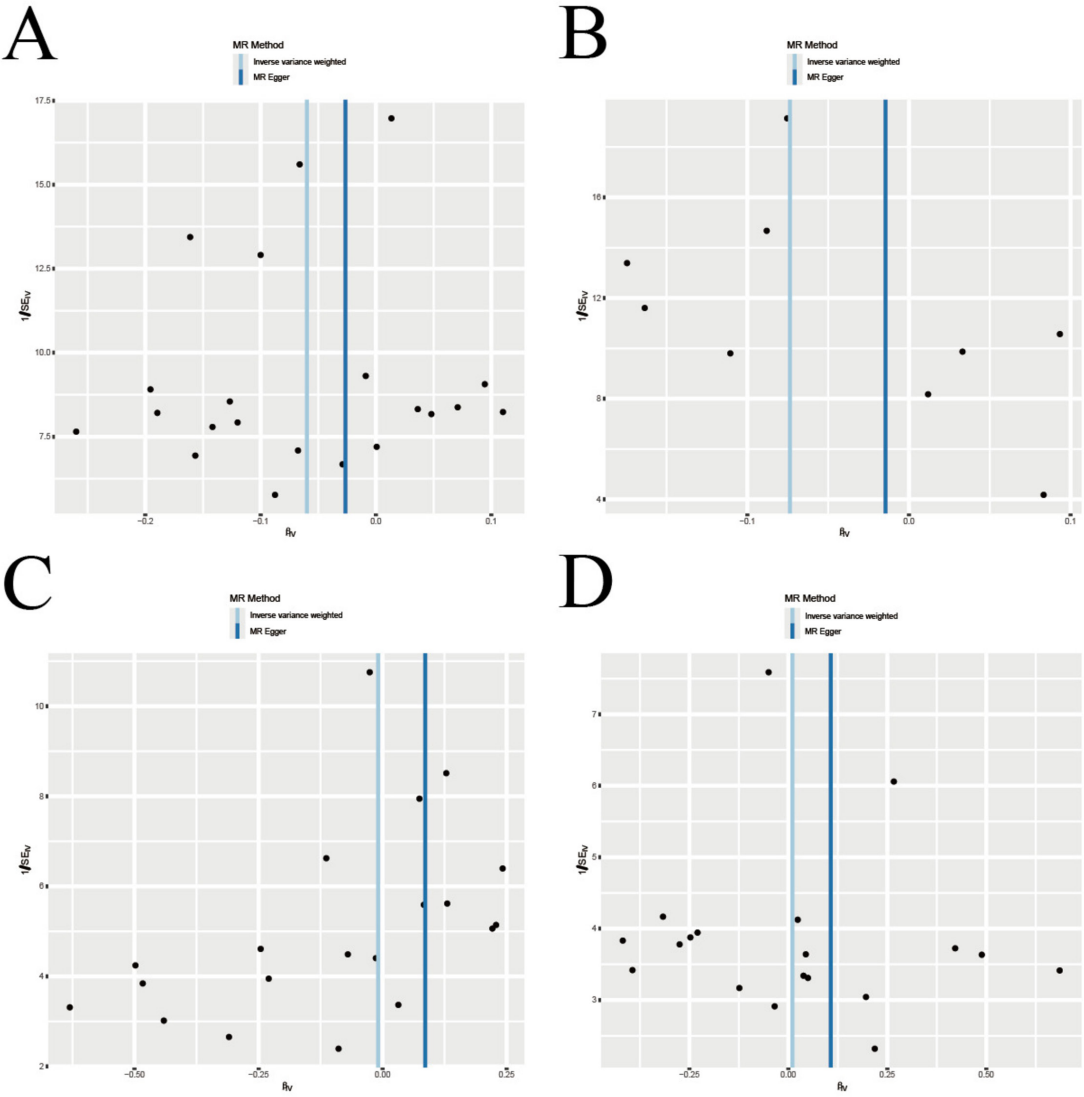
Funnel plots of bidirectional Mendelian randomization analyses. (A–B) correspond to forward MR analyses (Graves' disease and thyrotoxicosis with diffuse goitre, respectively); (C–D) correspond to reverse MR analyses with the same outcomes.

**FIGURE 6:**
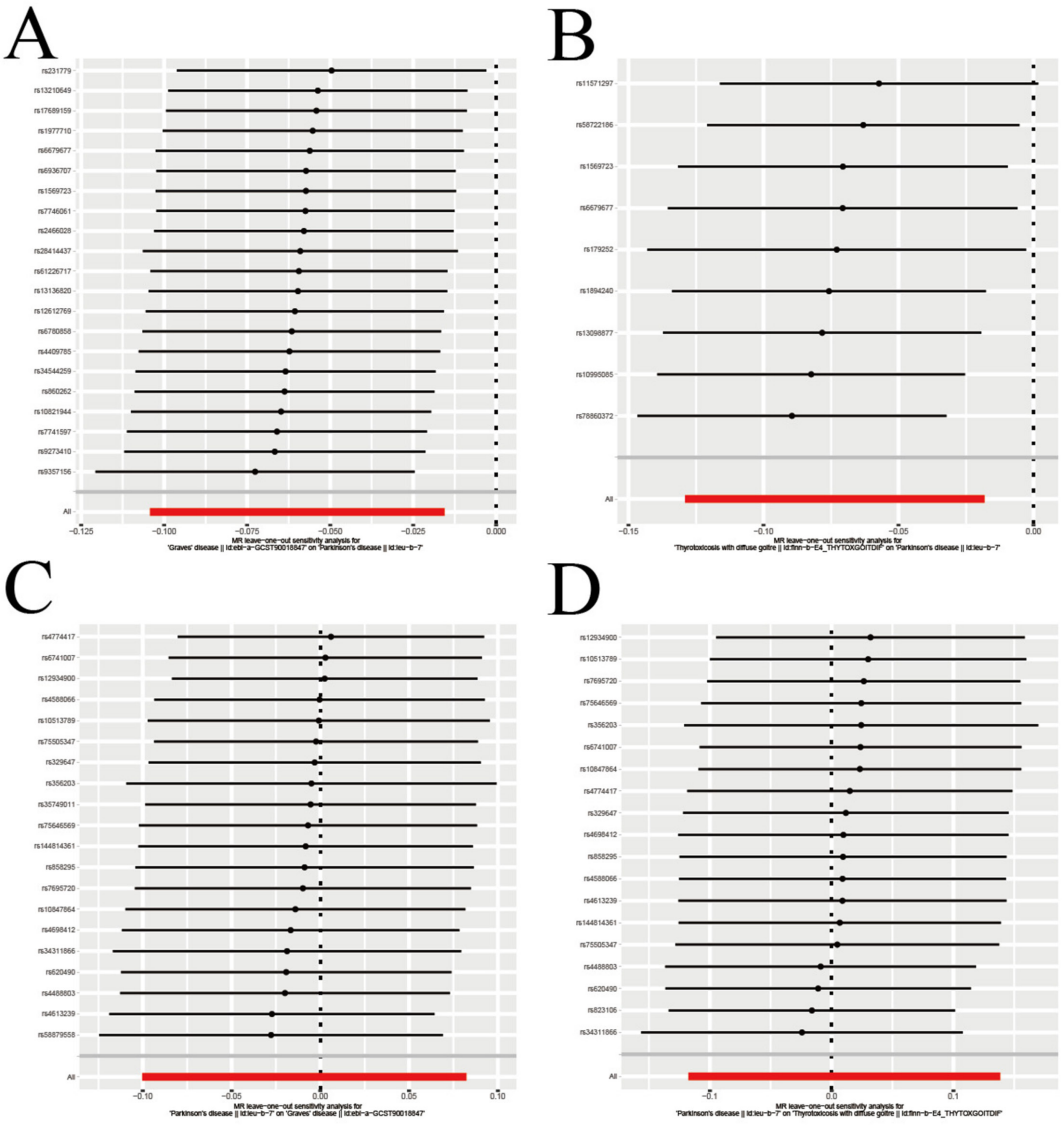
Leave‐one‐out sensitivity analyses of bidirectional Mendelian randomization results. (A–B) show forward MR analyses for Graves' disease and thyrotoxicosis with diffuse goitre, respectively; (C–D) show reverse MR analyses for the same outcomes.

Comprehensive sensitivity analyses were conducted to evaluate the reliability of the MR findings. As summarized in Table 1, Cochran's *Q* statistics for all forward and reverse MR analyses yielded *p*‐values of > 0.05, suggesting no significant heterogeneity among the IVs. The MR–Egger intercept tests and MR‐PRESSO global tests did not detect any horizontal pleiotropy or outlier SNPs, further supporting the robustness of the causal estimates. Visual inspection of the MR results through scatter plots (Figure 3), forest plots (Figure 4) and funnel plots (Figure 5) demonstrated the overall consistency and symmetry of the effect estimates across different analytical methods. The leave‐one‐out sensitivity analysis (Figure 6) revealed that sequential exclusion of individual SNPs did not materially alter the causal estimates, indicating that no single variant disproportionately influenced the overall association between hyperthyroidism diseases and PD risk.

**TABLE 1:** Assessment of heterogeneity and horizontal pleiotropy in bidirectional Mendelian randomization analyses.

Exposure	Outcome	Heterogeneity		Pleiotropy		Presso	
		*Q*	*Q*_pval	Intercept	*p*	RSSobs	*p*
Graves' disease	Parkinson's disease	17.045	0.650	−7.39E‐03	0.556	19.076	0.652
Thyrotoxicosis with diffuse goitre	Parkinson's disease	8.230	0.411	−1.63E‐02	0.560	10.059	0.470
Parkinson's disease	Graves' disease	24.896	0.164	−1.57E‐02	0.486	27.046	0.203
Parkinson's disease	Thyrotoxicosis with diffuse goitre	23.613	0.168	−1.50E‐02	0.638	26.377	0.175

For the multivariable MR (MVMR) models, sensitivity analyses also confirmed the stability of the results (Table 2). Across all adjusted models, Cochran's *Q* tests yielded nonsignificant results (*p* > 0.05), and MVMR–Egger regressions demonstrated no evidence of horizontal pleiotropy. These findings collectively underscore the robustness and reliability of the identified associations after adjustment for multiple potential confounders.

**TABLE 2:** Sensitivity analyses of multivariable Mendelian randomization models (MVMR).

Exposure	Confounding	Nsnp	*F*‐statistic	Heterogeneity		Pleiotropy	
				*Q*stat	*Qp*‐value	Estimate	*p*
Graves' disease	Smoking intensity	2	6.423	18.736	0.539	−1.39E‐03	0.814
	Adjusted by smoking	21	61.726	—	—	—	—
	Alcohol drinker	3	5.516	20.775	0.411	3.98E—05	0.997
	Adjusted by alcohol	20	62.444	—	—	—	—
	Body mass index	129	57.259	156.399	0.193	−6.50E—04	0.869
	Adjusted by BMI	16	9.431	—	—	—	—
Thyrotoxicosis with diffuse goitre	Smoking intensity	2	12.064	8.058	0.428	−4.90E—03	0.575
	Adjusted by smoking	9	46.209	—	—	—	—
	Alcohol drinker	3	9.421	14.338	0.111	2.08E—02	0.267
	Adjusted by alcohol	9	42.051	—	—	—	—
	Body mass index	132	58.718	150.156	0.192	−3.29E—04	0.938
	Adjusted by BMI	7	4.278	—	—	—	—

## 4 Discussion

In this MR study, we evaluated the bidirectional relationship between hyperthyroid disease states and PD using large‐scale genome‐wide association summary data from European populations. The analyses revealed consistent inverse associations between genetically predicted Graves' disease and thyrotoxicosis with diffuse goitre and the risk of PD. These associations remained stable after adjustment for smoking, alcohol consumption and BMI in MVMR analyses, and no reverse association was observed, indicating that PD liability does not measurably influence the genetic risk of these thyroid disorders. Taken together, these findings are consistent with a potentially protective relationship between hyperthyroid disease states and PD; we note, however, that—as discussed below—they should be interpreted with appropriate caution given the inherent assumptions of MR (Davies et al. 2018).

Previous observational studies of thyroid disorders and PD have reported inconsistent results. Some cohorts found hypothyroidism to be associated with a higher PD risk (Chen et al. 2020), whereas others reported null or even inverse associations (Berger and Kelley 1981). These discrepancies likely reflect residual confounding, small sample sizes and the difficulty of distinguishing causality from correlation in conventional epidemiological designs (Davies et al. 2018). Our MR findings provide genetic evidence consistent with hyperthyroid disease states being associated with a reduced risk of PD. This is in line with emerging evidence that thyroid hormones influence dopaminergic metabolism, mitochondrial activity and neuroinflammatory signalling—pathways central to PD pathogenesis (Cano‐Europa et al. 2008). Autoimmune thyroid disorders such as Graves' disease may further engage immune‐regulatory mechanisms that counteract chronic neuroinflammation (Antonelli et al. 2015; De Vito et al. 2011).

Our results should be considered alongside several recently published MR studies of thyroid–brain interactions. Zeng and colleagues, focusing on continuous thyroid function indices (TSH, free thyroxine), found no evidence of a causal link between thyroid function and PD in a bidirectional MR framework (Zeng et al. 2024). Wang and colleagues reported associations between thyroid function and prospective memory or dementia, with relatively weak signals for PD (Wang et al. 2025). Lei and colleagues, using bidirectional MR and co‐localization, observed evidence that PD liability may be protective against hypothyroidism but did not detect a reciprocal effect of hypothyroidism on PD (Lei et al. 2024). Schneider and colleagues, in a systematic review, summarized heterogeneous clinical observations linking thyroid dysfunction to a spectrum of movement disorders, while emphasizing the limited rigour of much of the available evidence (Schneider et al. 2023). Two features distinguish our analysis from these prior reports. First, we focused on clinically defined hyperthyroid disease states (Graves' disease and thyrotoxicosis with diffuse goitre) rather than continuous thyroid biomarkers; disease‐level genetic instruments capture composite effects of excess thyroid hormone signalling together with autoimmune and structural thyroid abnormalities that biomarker‐only exposures may underestimate. Second, we explicitly adjusted for major lifestyle confounders (smoking, alcohol, BMI) within MVMR models, addressing concerns that prior associations could be confounded by lifestyle differences in people with thyroid disease. Our findings should therefore be viewed not as overturning these earlier reports but as adding a complementary, disease‐centred dimension to a literature in which the directionality and magnitude of thyroid–PD relationships remain actively debated.

Several biological mechanisms may explain the observed protective associations. Thyroid hormones are critical regulators of neuronal differentiation, synaptic transmission and mitochondrial energy metabolism (Bernal 2017; Talhada et al. 2019). Dysregulation of thyroxine (T₄) and triiodothyronine (T₃) levels can influence oxidative‐stress responses and dopaminergic neuron integrity (Cano‐Europa et al. 2008; Rahaman et al. 2001). Enhanced thyroid hormone signalling may promote mitochondrial biogenesis and antioxidant capacity, thereby attenuating dopaminergic neurodegeneration (Weitzel and Iwen 2011; Lin et al. 2020). Autoimmune thyroid disease may also exert neuroprotective effects through immune modulation (Jara et al. 2017): Increased regulatory T‐cell activation or altered cytokine profiles in autoimmune thyroiditis could attenuate microglial activation and reduce neuroinflammatory stress (González and Pacheco 2014). Thyroid hormone signalling may additionally influence α‐synuclein aggregation and clearance, potentially mitigating the pathological protein accumulation that characterizes PD (Lin et al. 2020; Decressac et al. 2013). Although these mechanisms remain hypothetical, they provide biologically plausible explanations for the observed associations.

Although the present analyses focused on PD, clinically, there are well‐recognized links between thyroid dysfunction and a broader spectrum of movement disorders. The systematic review by Schneider and colleagues documented associations between hyperthyroidism and chorea, postural and action tremor, myoclonus and parkinsonism‐like phenomena, whereas hypothyroidism has been described in association with cerebellar ataxia and bradykinesia (Schneider et al. 2023). Whether the protective genetic relationship we observed extends to these phenotypes is unknown, in part because robust, well‐powered GWAS for many of these rarer movement disorders are not yet available. Extending MR analyses to a wider range of movement disorders, as adequate genetic data accumulate, would help to clarify the scope of the thyroid–brain axis in motor neurodegeneration and to test whether the inverse association identified here is PD‐specific or reflects a broader neuroprotective influence of hyperthyroid pathophysiology.

From a clinical perspective, our findings highlight thyroid function as a potentially relevant axis to consider in PD research and risk stratification. Because hyperthyroid disease states were genetically associated with lower PD risk, thyroid hormone homeostasis and thyroid autoimmunity may influence neurodegenerative trajectories. We emphasize, however, that our results do not justify any change in clinical practice and in particular do not support thyroid hormone administration or alteration of thyroid management for PD prevention. Rather, they support continued investigation of thyroid status and thyroid‐related autoimmunity as contributors to neurodegenerative susceptibility, and they motivate carefully designed mechanistic, longitudinal and—where appropriate—interventional studies before any translational application can be considered.

The present study has several notable strengths. It represents one of the largest disease‐level MR analyses of hyperthyroid disorders and PD to date, drawing on high‐quality GWAS data from FinnGen and IEU Open GWAS. The bidirectional and MVMR design reduces vulnerability to confounding and reverse causation that affects conventional observational designs, and our analyses included adjustment for major lifestyle confounders (smoking, alcohol, BMI). Several limitations should, however, be acknowledged. First, the analyses were restricted to individuals of European ancestry. We searched East Asian and trans‐ancestry GWAS sources for hyperthyroid disease states and for PD, but currently available non‐European datasets either provided null or underpowered signals, lacked publicly accessible summary statistics with sufficient quality control or yielded too few genome‐wide significant instruments for valid MR. As such datasets mature, replication in East Asian and other non‐European populations will be an important priority, both to confirm the present findings and to evaluate ancestry‐related differences in the thyroid–brain axis. Second, we focused on two clinically prominent hyperthyroid entities; other less common thyroid phenotypes and movement disorders beyond PD were not analysed and represent natural extensions of this work as data become available. Third, although MR provides stronger inferential support than conventional observational designs, it does not by itself establish biological causality: the genetic instruments index lifelong, modest perturbations of the exposure, and the core assumptions of relevance, independence and exclusion restriction can only be partially verified. The inverse associations observed here should therefore be interpreted as supportive—but not definitive—evidence for a protective role of hyperthyroid pathophysiology in PD. Fourth, the analyses were based on summary‐level data and could not be stratified by age, sex, or disease stage. Finally, the use of registry‐ and self‐report‐derived phenotypes inherent to large biobank GWAS may introduce misclassification; although our sensitivity analyses indicated no horizontal pleiotropy or heterogeneity, the possibility of unrecognised biological pleiotropy cannot be fully excluded. Abbreviations have been standardized across the manuscript and listed at the end.

In conclusion, this MR study provides genetic evidence consistent with a protective association between hyperthyroid disease states (Graves' disease and thyrotoxicosis with diffuse goitre) and PD, independent of major lifestyle factors. These results should be regarded as hypothesis‐strengthening rather than definitively causal, but they highlight the thyroid–brain axis—including thyroid hormone signalling and immune‐mediated mechanisms—as a biologically plausible and potentially modifiable contributor to PD risk. Replication in non‐European populations, extension to a wider spectrum of movement disorders and mechanistic validation will be essential before these findings can be translated into preventive or therapeutic insight.

## Author Contributions

**Meng Luo:** conceptualization, methodology, formal analysis, investigation, data curation, writing – original draft. **Shiren Huang:** methodology, formal analysis, validation, writing – review and editing. **Wei Wang:** conceptualization, supervision, project administration, writing – review and editing. All authors reviewed and approved the final manuscript. Contributions are described using the CRediT taxonomy.

## Ethics Statement

This study is a Mendelian Randomization analysis based on previously published data and therefore did not require ethical approval or patient consent.

## Conflicts of Interest

The authors declare no conflicts of interest.

## Supporting information


**Table S1:** Summary of GWAS datasets used in the Mendelian randomization analysis.
**Table S2:** List of instrumental variables (SNPs) included in the Mendelian randomization analyses.

## Data Availability

The GWAS summary data analysed in this study are publicly available from the FinnGen consortium (https://www.finngen.fi/en) and the IEU Open GWAS Project (https://gwas.mrcieu.ac.uk/). Further details are available from the corresponding author upon reasonable request.
