# Supplementary material for: Hyperthyroidism Is Genetically Associated With Reduced Risk of Parkinson's Disease: A Mendelian Randomization Analysis
Source: Eur J Neurosci. 2026 Jul 8;64(1):e70616. doi: 10.1111/ejn.70616 (PMC13344109; doi:10.1111/ejn.70616)
Supplement: Supplementary file 1 — Table S1: Summary of GWAS datasets used in the Mendelian randomization analysis. Table S2: List of instrumental variables (SNPs) included in the Mendelian randomization analyses. [file EJN-64-0-s001.pdf]

Supplementary Table S1. Summary of GWAS datasets used in the Mendelian randomization analysis.

	Trait	Dataset	Year	Race	Sample Size	nSNP
**Outcome**	Parkinson's disease	ieu-b-7	2019	European	482730	17891936
**Exposures**	Graves' disease	ebi-a-GCST90018847	2021	European	458620	24189816
	Thyrotoxicosis with diffuse goitre	finn-b-E4_THYTOXGOITDIF	2021	European	190034	16380368
**Confoundings**	Smoking intensity (cotinine levels/cigarettes per day)	ebi-a-GCST009966	2020	European	4772	8648245
	Alcohol drinker status: Current	ukb-d-20117_2	2018	European	360726	13586591
	Body mass index	ebi-a-GCST006368	2018	European	315347	27854527

Supplementary Table S2. List of instrumental variables (SNPs) included in the Mendelian randomization analyses.

	**SNP**	**CHR**	**POS**	**Effect Allele**	**Other Allele**	**BETA**	**EAF**	**SE**	**Pval**	**Sample size**	**R2**	**F**
	rs10821944	10	63785089	T	G	-0.157	0.703	0.023	1.43E-11	458620	9.93E-05	45.56
	rs12612769	2	191953998	C	A	0.145	0.218	0.025	8.78E-09	458620	7.21E-05	33.06
	rs13136820	4	40307564	T	C	-0.136	0.700	0.024	2.37E-08	458620	6.78E-05	31.11
	rs13210649	6	167473193	G	T	0.135	0.442	0.022	6.87E-10	458620	8.33E-05	38.22
**Graves' disease**	rs1569723	20	44742064	A	C	0.164	0.712	0.023	1.62E-12	458620	1.09E-04	50.03
	rs17689159	16	79742390	C	T	0.162	0.305	0.024	1.55E-11	458620	9.95E-05	45.62
	rs1977710	1	157693722	G	A	0.146	0.444	0.022	5.19E-11	458620	9.36E-05	42.92
	rs231779	2	204734487	T	C	0.235	0.468	0.022	3.61E-26	458620	2.45E-04	112.24
	rs2466028	8	128200556	C	T	-0.145	0.361	0.02	6.28E-1	458620	8.30E-05	38.07

							4	0			
rs28414437	14	81457257	C	A	0.287	0.424	0.023	1.98E-36	458620	3.46E-04	158.56
rs34544259	21	43834854	G	A	0.141	0.405	0.023	6.75E-10	458620	8.29E-05	38.03
rs4409785	11	95311422	C	T	0.203	0.144	0.035	6.81E-09	458620	7.33E-05	33.61
rs61226717	12	111315543	T	G	0.237	0.109	0.036	3.60E-11	458620	9.57E-05	43.89
rs6679677	1	114303808	A	C	0.377	0.112	0.054	2.14E-12	458620	1.08E-04	49.44
rs6780858	3	188132110	G	A	-0.163	0.468	0.023	3.85E-13	458620	1.15E-04	52.74
rs6936707	6	32684069	T	C	-0.463	0.035	0.048	4.61E-22	458620	2.03E-04	93.31
rs7741597	6	29701661	C	T	-0.222	0.144	0.029	2.27E-14	458620	1.27E-04	58.34
rs7746061	6	31419216	A	G	-0.384	0.056	0.052	1.02E-13	458620	1.20E-04	55.27
rs7754251	6	90989125	C	G	0.131	0.490	0.023	7.16E-09	458620	7.28E-05	33.41

	rs860262	7	28194397	A	C	-0.137	0.418	0.025	2.11E-08	458620	6.85E-05	31.41
	rs9273410	6	32627250	A	C	0.307	0.429	0.024	1.52E-37	458620	3.57E-04	163.84
	rs9357156	6	33041073	C	A	0.440	0.231	0.025	1.09E-68	458620	6.68E-04	306.74
	rs10995085	10	63961173	T	C	0.211	0.227	0.037	8.72E-09	190034	1.74E-04	33.12
	rs11571297	2	204745003	C	T	-0.228	0.374	0.031	3.57E-13	190034	2.78E-04	52.88
	rs13098877	3	188133163	T	C	0.186	0.521	0.030	1.03E-09	190034	1.96E-04	37.27
**Thyrotoxicosis with diffuse goitre**	rs1569723	20	44742064	A	C	0.188	0.729	0.034	4.56E-08	190034	1.57E-04	29.93
	rs179252	14	81435985	G	T	-0.346	0.590	0.031	8.67E-29	190034	6.52E-04	124.06
	rs1894240	22	23052772	G	A	-0.223	0.513	0.032	3.07E-12	190034	2.56E-04	48.65
	rs58722186	16	79751591	T	C	0.215	0.309	0.033	7.01E-11	190034	2.24E-04	42.59
	rs6679677	1	114303808	A	C	0.428	0.143	0.04	1.22E-2	190034	4.81E-04	91.44

								5	1			
	rs78860372	6	30380366	T	A	0.406	0.144	0.062	4.48E-11	190034	2.28E-04	43.40
	rs10451230	17	16035225	T	A	-0.096	0.565	0.018	4.42E-08	482730	6.23E-05	30.09
	rs10513789	3	182760073	G	T	-0.160	0.183	0.022	3.18E-13	482730	1.10E-04	53.11
	rs10847864	12	123326598	T	G	0.127	0.363	0.018	9.81E-13	482730	1.05E-04	50.66
	rs12934900	16	30923602	T	A	0.122	0.657	0.018	4.33E-11	482730	9.03E-05	43.60
**Parkinson's disease**	rs144814361	10	121410917	T	C	0.441	0.017	0.068	9.07E-11	482730	8.72E-05	42.08
	rs329647	11	133764666	C	G	-0.113	0.666	0.018	1.94E-10	482730	8.39E-05	40.52
	rs34311866	4	951947	C	T	0.227	0.196	0.023	7.97E-23	482730	2.00E-04	96.74
	rs356203	4	90666041	T	C	-0.240	0.617	0.018	3.01E-41	482730	3.76E-04	181.49
	rs35749011	1	155135036	A	G	0.751	0.019	0.06	5.02E-3	482730	2.69E-04	129.80

							6	0			
rs4488803	3	58218352	A	G	-0.114	0.375	0.020	1.08E-08	482730	6.75E-05	32.59
rs4588066	18	40672964	A	G	0.105	0.326	0.018	4.45E-09	482730	7.15E-05	34.53
rs4613239	2	169119609	G	C	0.178	0.133	0.025	6.21E-13	482730	1.07E-04	51.75
rs4698412	4	15737348	A	G	0.126	0.553	0.017	7.05E-14	482730	1.16E-04	56.07
rs4774417	15	61993702	A	G	0.105	0.740	0.019	4.63E-08	482730	6.22E-05	30.02
rs58879558	17	44095467	C	T	-0.238	0.223	0.025	1.36E-21	482730	1.88E-04	90.86
rs620490	8	16697579	G	T	-0.117	0.276	0.019	6.46E-10	482730	7.91E-05	38.18
rs6741007	2	135537119	G	T	-0.123	0.451	0.018	2.09E-12	482730	1.03E-04	49.64
rs75505347	12	40885549	T	C	0.392	0.020	0.067	6.12E-09	482730	7.00E-05	33.77
rs75646569	5	60345424	G	T	0.192	0.112	0.02	5.62E-1	482730	1.07E-04	51.88

							7	3			
rs7695720	4	77183300	C	A	-0.126	0.209	0.021	1.53E-09	482730	7.54E-05	36.40
rs823106	1	205656453	C	G	-0.149	0.849	0.024	4.10E-10	482730	8.07E-05	38.97
rs858295	7	23245569	G	A	-0.104	0.395	0.018	3.83E-09	482730	7.22E-05	34.85

2
